# X-Ray absorption spectroscopy on airborne aerosols

**DOI:** 10.1039/d2ea00016d

**Published:** 2022-10-03

**Authors:** Muhammad H. Rashid, Camelia N. Borca, Jacinta M. Xto, Thomas Huthwelker

**Affiliations:** Paul Scherrer Institute, Swiss Light Source, Laboratory for Femtochemistry Forschungsstrasse 111 Villigen PSI Switzerland Thomas.Huthwelker@psi.ch

## Abstract

Here we demonstrate a method for performing X-ray absorption spectroscopy (XAS) on airborne aerosols. XAS provides unique insight into elemental composition, chemical and phase state, local coordination and electronic structure of both crystalline and amorphous matter. The aerosol is generated from different salt solutions using a commercial atomizer and dried using a diffusion drier. Embedded in a carrier gas, the aerosol is guided into the experimental chamber for XAS analysis. Typical particle sizes range from some 10 to a few 100 nm. Inside the chamber the aerosol bearing gas is then confined into a region of about 1–2 cm^3^ in size, by a pure flow of helium, generating a stable free-flowing stream of aerosol. It is hit by a monochromatic X-ray beam, and the emitted fluorescent light is used for spectroscopic analysis. Using an aerosol generated from CaCl_2_, KCl, and (NH_4_)_2_SO_4_ salt solutions, we demonstrate the functionality of the system in studying environmentally relevant systems. In addition, we show that the detection limits are sufficient to also observe subtle spectroscopic signatures in XAS spectra with integration times of about 1–2 hours using a bright undulator beamline. This novel setup opens new research opportunities for studying the nucleation of new phases in multicomponent aerosol systems *in situ*, and for investigating (photo-) chemical reactions on airborne matter, as relevant to both atmospheric science and also for general chemical application.

## Abstract 2

It is demonstrated that X-ray absorption spectroscopy can be used to analyze the chemical state of airborne aerosols *in situ* under controlled conditions.

## Graphical abstract

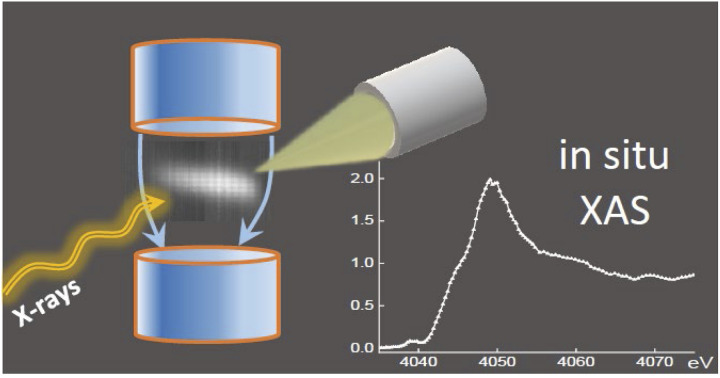

**Environmental significance**

Atmospheric aerosols are complex mixtures of organics, electrolyte solutions or solid inorganic salts. They serve as catalysts in the atmosphere, contribute to photochemical processes, and are nuclei for cloud formation. Model experiments are key in deciphering the environmental functionality of aerosols. Experiments with airborne aerosols mimic natural environmental processes better than those using bulk matter. They are also advantageous because there is no sample-container contact, which could induce undesired chemical reactions or heterogeneous nucleation on sample container walls. However, spectroscopy on airborne matter is a significant analytical challenge, because the total amounts of material is very small. Here, we demonstrate that X-ray absorption spectroscopy, which can record electronic structure, oxidation state, local coordination and phase state, can be used for spectroscopic analysis of airborne matter. The presented new method paves an avenue to novel model studies for atmospherically relevant (photo-) chemical processes on aerosols, their formation, or for the nucleation of new phases, and hence their environmental impact.

## Introduction

A myriad of chemical and physical process in the atmosphere involve micrometer-sized airborne particles, such as the destruction of stratospheric ozone, which is catalyzed by icy particles.^1,2^ Micrometer-sized particles also serve as nucleation sites for the formation of droplets and ice in clouds,^3–5^ and hence are key to the Earth's radiation balance^6^ and climate change. In addition, mixed organic aerosols also participate in tropospheric radical chemistry affecting levels of reactive oxygen species, and hence influencing human health.^7^ The main inorganic constituents of atmospheric aerosols are elements which are lighter than iron (*i.e.* O–Fe), including halogens, such as chlorine and bromine, or mixed organic sea-salt aerosols, with a significant aqueous fraction of sodium, chlorine and sulfate.^8,9^ These elements can be found as ions in aqueous solutions, as precipitated crystallites, or embedded in organic matter. The chemical and physical state of these materials in a specific system then defines their chemical and physical function in the atmosphere.

Due to the microscopic size of aerosols particles, their chemical behavior differs from bulk material, which can affect the radical chemistry^10^ and photochemistry in aerosol droplets and particles.^11^ In addition, microscopic airborne droplets can be useful in studying supersaturated matter. In a multicomponent aerosol droplet, nucleation occurs either by homogeneous or heterogeneous nucleation. The latter is seeded by contact of supersaturated matter with another interface, such as gas–liquid, liquid–solid, or other liquid phases or minerals, as it has been discussed for the nucleation of ice from aqueous solutions.^12^ Nucleation studies are often hampered by unwanted heterogeneous nucleation on the walls of the sample container which can induce the nucleation of a new phase. This problem can be overcome by performing experiments on airborne particles, such as experiments in a smog chamber,^13^ or in electrodynamic balances.^14–17^

Several approaches have been used to study aerosol particles, for a recent review see Ault and Axson.^18^ For elemental analysis on collected aerosols X-ray fluorescence spectroscopy^19,20^ has been used. The development of X-ray scanning transmission microscopy (STXM)^21,22^ with spatial resolution in the 20 nm scale allowed for the first time to characterize the morphology, internal physical mixing state, and the chemical state of individual aerosol particles.^23^ This technique has been used to study the role of aerosols for climate forcing,^24^ the iron speciation in mixed aerosol particles,^25^ and chemical state of the organic phase,^26^ the role of aerosols for ice nucleation^27,28^ or radical chemistry.^10^ In addition, the development of different *in situ* reactors,^29–33^ has allowed chemical *in situ* studies for catalysis,^34^ electrochemistry^35^ and environmental science, such as measuring the uptake of water on individual salt aerosol or soot,^36,37^ phase separation in multiphase aerosols,^38^ or linking the aerosol morphology to photochemical processes.^10,11^

One step towards the study of airborne matter employed the generation of clusters and nanoparticles^39,40^ for investigations using electron velocity imaging (VMI). This technique has been further developed and adapted to study chemical reactions in aqueous and organic systems^41–43^ using a differentially pumped inlet. Pioneered by researchers at the ALS and Bessy,^44,45^ X-ray photoelectron spectroscopy (XPS) has been coupled with a liquid microjet of an aqueous solution, or a droplet train system.^46^ One important rationale to use the very surface sensitive XPS system is to decipher structural differences between bulk and surface molecules in solutions, as it has been first demonstrated by Ghosal and co-workers.^47,48^

Liquid jet or droplet train experiments employ samples which are sized in order of some 10 μm, which is large compared to the submicrometer-sized atmospheric aerosol particles. To directly study aerosol particles using XPS, two synchrotron beamlines (the chemical dynamics beamline at the Advanced Light Source^49^ and Pleiades at Soleil^50^) implemented an aerodynamic lens to introduce a focused aerosol jet into a low-pressure chamber for analysis with soft X-ray photoemission spectroscopy. In these setups, aerosol particles were synthesized and chemically processed under controlled conditions, and afterwards brought into vacuum, as required for XPS analysis. Using this setup, Ouf *et al.*^51^ investigated the carbon K edge with both Near Edge X-ray Absorption Spectroscopy (NEXAFS) and XPS techniques from freshly emitted carbon soot in the aerosol phase. Abid *et al.*^52^ studied CaCl_2_ nanoparticles and demonstrated that the first coordination shell for the Ca^+^ and Cl^−^ ions remains similar in hydrated CaCl_2_-NPs, but differs significantly from the one in aqueous solution and solid CaCl_2_. Jacobs and co-workers studies found chemical gradients in the organic fraction of airborne particles.^53^ The aerodynamic lens approach is also used at the X-ray free electron laser facilities for single particle imaging.^54–56^

Exploiting the surface sensitivity of XPS provides important information about the aerosol particle surface. However, given the fast liquid phase diffusion constants also bulk information are relevant to assess the chemical reactivity of mixed phase aerosols. Moreover, when using an aerodynamic lens, the pressure drop along the aerodynamic lens, imposes rapid thermodynamic changes to the aerosol when passing the lens. XAS can provide important complementary bulk information if performed at ambient pressures under well-controlled thermodynamic conditions.

XAS, which is typically performed at synchrotrons, measures the absorption of photons and the local scattering of photoelectrons around an excited atom. Therefore, XAS probes the electronic structure, local coordination, and hence the chemical phase state. It can also be used for the study of amorphous matter, as it probes short-range order. XAS is element-specific, and it is ideal for measuring hydrated elemental ions (*e.g.* Cl^−^, Ca^2+^, Na^+^, *etc.*). Being a mostly non-destructive technique, XAS is frequently used in catalysis,^57,58^ battery research^59^ and environmental science.^60,61^ For applications in these fields elements, lighter than Fe and transition metals are important. The absorption edges of these elements are in the rarely served energy range between 0.4 and 8 keV, *i.e.* mostly in the tender (1–5 keV) and soft X-ray range (<2 keV).

Probably, the first (and only) spectroscopic investigation of in-flight nanoparticles using XAS was demonstrated by Landron *et al.*^62^ using ultrasonically produced zirconia precursors and recording XAS spectra at the Zr K-edge (∼18 keV). These experiments were made in a larger cell, and the authors could distinguish between different coordination environments in airborne zirconium crystals. The spectroscopic study of airborne particles is hampered by the extremely small amount of condensed material in the gas phase, as further detailed in the Methods section. Besides the challenge of very low amounts of material, there are additional difficulties for such measurements, such as the need to either work under a helium atmosphere, or under vacuum, to control the thermodynamic state in the measurement region, and to prevent contaminations due to deposition of airborne matter in the experiment.

Herein X-ray absorption spectroscopy is used to study airborne aerosols generated by a commercial nebulizer, which are confined using a particle free gas into a stable nanoparticle stream. We demonstrate the functionality of the setup using aerosols generated from different inorganic salts with distinct deliquescence humidities and consisting of atmospherically relevant ions, such as SO_4_^2−^, Cl^−^, K^−^, Ca^2+^. We further discuss possible future applications relevant to atmospheric science and other chemistry fields.

## Methods

### X-ray absorption spectroscopy (XAS)

X-ray absorption spectroscopy (XAS) is an important analytical tool for chemical model studies. A XAS spectrum measures the X-ray absorption coefficient as function of the incoming photon's energy. Depending on its energy, the absorbed photon excites an inner-shell electron either into an unoccupied orbital, into a continuum state, or as photoelectron into vacuum. XAS spectra reflect directly the electronic structure of the probed atom, and identify its chemical bonding or oxidation state. Moreover, as photoelectrons scatter from the local atomic environment of the excited atom, XAS also a probes the local coordination environment. Once a photon is absorbed, the atom is in an excited or an ionized state with an electron–hole in an inner shell orbital. Then, the atom will decay back into the ground state by electronic transition of an electron into the empty inner-shell electron–hole. For example, an electronic transition from the L to the innermost K shell, will gives rise to the X-ray emission Kα line. The electronic transition into the ground state can also lead to the emission on of an Auger electron. Measuring the transmission of photons through matter is the most direct measurement of the absorption coefficient. It is proportional to both the number of fluorescent photons, and the generated photoelectrons. Therefore, XAS spectra can also be measured by recording the total electron yield (TEY), which is given by the intensity of all emitted electrons, or by the X-ray emission line as function of the excitation energy. The X-ray fluorescence can be measured in air, if the photon absorption in the gas phase is not too high: for example, the transmission through 10 cm of air at 2 keV is less than 1%, but it is 99% through 10 cm helium. Hence, in the soft and tender X-ray range, it is advantageous to place the sample either in vacuum or in helium, as we do in the setup presented below. The total electron yield is mostly measured in vacuum, by simple detection of the electrical current into the sample, which measures the loss of electrons. This detection mode can also be implemented in an helium atmosphere, if the charge transport through the gas phase is well controlled.^63^ A related detection method is X-ray photo electron spectroscopy (XPS), which analyzes the kinetic energy of the emitted photoelectrons. XPS provides a direct picture of the electronic and chemical structure of elements in the surface region with a surface sensitivity in the sub nm range for soft X-rays. Total electron yield records all electrons including Auger electrons and therefore probes a somewhat thicker surface layer in the order of 1–10 nm for sub keV photons.^64,65^ In contrast, fluorescence detection probes bulk matter. Its probing depth is energy and material dependent. In the soft and tender X-ray range, the typical penetration depth in water ranges from a few 100 nm (at 200 eV) to 230 μm at 5 keV.

As analytical method, XAS is sensitive to very small amounts of material, in particular in the soft X-ray range. This can be directly concluded from the characteristic absorption length. Depending on the photon energies and materials, the probing depth in fluorescence mode ranges from the submicrometer range for soft X-ray up to some 100 μm for tender X-rays.

### PHOENIX beamline

X-ray absorption spectroscopy measurements were performed at the PHOENIX (PHOtons for the Exploration of Nature by Imaging and XAFS) beamline at the Swiss Light Source (SLS, Villigen PSI), which covers both the soft and tender X-ray range (0.3 to 8 keV). This energy range is rarely served: there are only two main instruments in Europe: the LUCIA beamline (0.5–8 keV) at the Synchrotron Soleil^66^ and the PHOENIX beamline^67^ (0.3–8 keV) at the Swiss Light Source (SLS). An elliptical undulator serves as photon source for linear and elliptically polarized photons. There are two branchlines: PHOENIX II covers the soft X-ray range (0.3–2 keV) and PHOENIX I covers higher energies (1–8 keV), which include the tender X-ray range. PHOENIX I employs a double crystal monochromator for the high energy branchline (Double Crystal Monochromator, DCM 1–8 keV). A planar grating monochromator serves the soft X-ray endstation, which is located at the exit of the X-Treme beamline.^68^ Both branchlines offer either an unfocused beam (PHOENIX I: 1.5 × 1.5 mm, PHOENIX II 3 × 5 mm) or use a Kirkpatrick–Baez (KB) mirror system to focus the beam (PHOENIX I 2.5 × 2.5 μm, PHOENIX II 4 × 5 μm). To reduce the absorption of X-rays in the gas phase, the experimental endstation can be operated either under vacuum or under helium pressure of up to 800 mbar.

### Aerosol generation

**Fig. 1:**
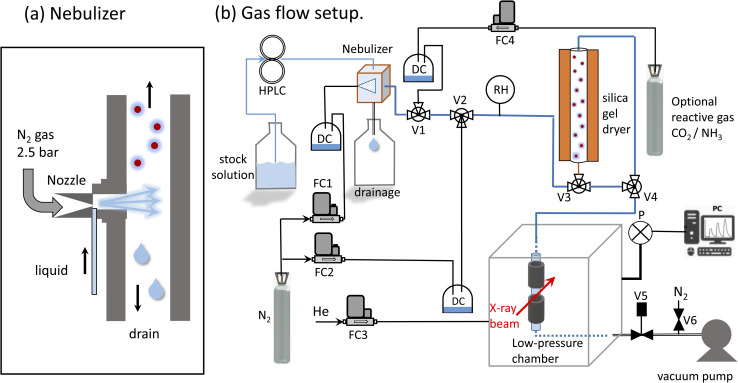
Experimental setup for the generation of aerosols. (a) Aerosols are generated using a commercial nebulizer, which is operated using compressed nitrogen. (b) Schematic of the flow system for aerosol generation and coupling to the endstation. HPLC: high performance liquid chromatography pump, DC: droplet collector to prevent contamination of mass flow controllers, V1: 3-way valve to add reactive gases, V2: option to add additional N_2_ to reduce humidity, V3/V4: valves to switch dryer on/off, V5: regulation valve to regulate pressure in endstation, V6: needle valve for additional N_2_ flow to reduce humidity in vacuum pump, P: vacuum gauge, RH: humidity sensor, FC1: gas flow controller for nebulizer, FC2: gas flow controller to change humidity level, FC3: gas flow controller for backflow into chamber. An additional aerosol filter (not shown) protects the vacuum pump from contamination with particles.

The system developed by Xto *et al.*^69^ was slightly adapted to generate a continuous aerosol stream consisting of salt solution droplets. A commercial nebulizer (TSI model 3076, Fig. 1a) was employed to nebulize a salt solution through an orifice (0.35 mm) into a flow of dry nitrogen. The nitrogen pressure at the nebulizer inlet was 2–2.5 bars, and the optimal flow rate was 1.8 l min^−1^. The liquid was pumped into the nebulizer by a HPLC pump (manufacturer: Watrex, model P102) with liquid flow rates of 0.7 to 1.1 ml min^−1^. Flow parameters were chosen to maximize the amount of nebulized liquid. For a liquid flow rate of 0.6 ml min^−1^, the nebulizer is designed to produce about 2 × 10^6^ particles per cm^3^ with a mean diameter of 0.3–0.4 μm.^70^ The relative humidity at the nebulizer's exit was between 80 and 90%. The humidity was not monitored continuously during the experiment, because we observed that the humidity sensor corrodes upon prolonged deposition of salt droplets. Any excess liquid was drained from the sprayer and collected in the reservoir below. The aerosol carrying gas stream was then led into a gas flow system for further processing in a dryer. The dryer was composed of a bed of silica gel of 50 cm length and 6 cm diameter. The semi-permeable wick located in the interior allows the exchange of humidity between the gas and the silica gel. At the exit of the dryer the relative humidity was close to 10–30%. To minimize electrostatic effects, which can lead to aerosol loss to the walls, electrically conducting tubing (6 mm outer diameter) and metal fittings were used (Swagelock).

### Implementation of a confined free-flowing aerosol stream in a low-pressure chamber

**Fig. 2:**
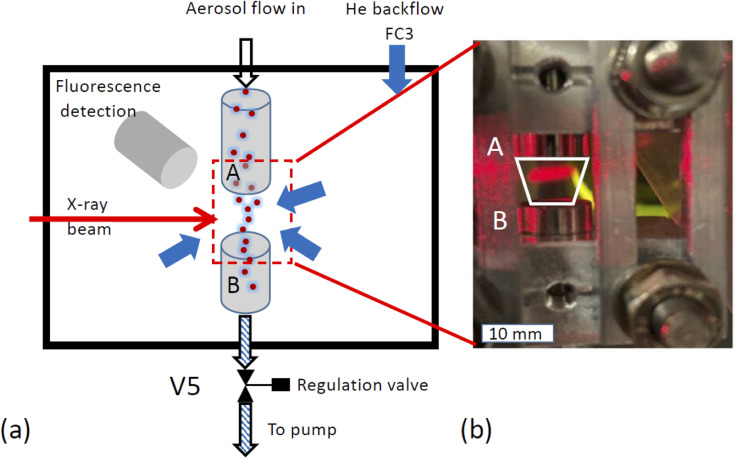
Implementation of a free-flowing stream of aerosol in the endstation (a) sketch of setup. A: inlet for carrier gas with aerosol, B: pumped outlet which pumps all gases and the aerosol out of the chamber, inner diameter of both inlet and outlet: 10 mm, V5: regulation valve, FC3: He backfill gas flow, blue arrows symbolize that the backfill gas, serves as confinement by exerting an isotropic pressure to the free-flowing aerosol stream. Both aerosol and backfill gas are pumped through the same outlet B. (b) Photograph of free-flowing aerosol stream in the low-pressure chamber. The white trapezoid marks region to which the aerosol is confined. Part of the aerosol stream is illuminated by a red laser beam.

X-ray spectroscopy was performed on the airborne particle by confining the aerosol stream with an inert gas. The concept is illustrated in Fig. 2a. The carrier gas with the aerosols is guided into the experimental endstation *via* an open metal tube (A) and pumped out of the endstation *via* outlet B together with all gases entering the endstation. Using this arrangement, the particle free He backflow confines the airborne particle stream into a small volume between inlet A and outlet B, preventing any contamination of the experimental endstation with aerosol particles.

The functionality of the system is demonstrated in Fig. 2b, which shows a photo of the set up mounted in the experimental chamber of the beamline. The trapezoid shows the region where the aerosol flow is confined between inlet A and outlet B. Here, a laser diode illuminates a part of the region between the in- and outlets where the aerosol reflects the laser beam. This demonstrates that the aerosol flow is nicely confined to the region between inlet and outlet.

### Pressure regulation

For a reliable operation of the setup, both gas flows and pressure in the chamber must be kept very stable, and were therefore controlled by mass flow controllers (manufacturer: Bronkhorst). The pressure in the endstation was regulated by computer controlled needle valve (manufacturer: Pfeiffer, type RME-005A) at the exit of outlet B to keep the chamber pressure constant. To protect this valve from clogging with aerosols, a metal tube (38 mm inner diameter, 15 cm length) filled with household cotton wool, was placed before the regulation valve. Using this method, the pressure in the chamber can be stabilized within 0.1% in the range of 600–800 mbar.

### SiN window

The beamline, operating in high vacuum (<10^−7^ mbar), is separated from the endstation by a silicon nitride window (0.5 μm thick), which serves as main gas barrier. To protect this window, a second safety barrier, namely a Kapton foil (7 micron thickness, about 80% transmission at 3 keV) was mounted directly over the window. In case the silicon nitride window breaks, the Kapton foil would be pressed onto the frame of the silicon nitride window by the gas in the endstation. This would slow down the venting of a small chamber between beamline optics and endstation, leaving time to close the additional automated shutter to the beamline, and providing additional safety to protect the beamline from contamination with aerosols.

### SDD detector

The emitted fluorescence from the aerosols was measured using a silicon drift detector (SDD, manufacturer Ketek, Germany). The detector is mounted in a 90 degree angle relative to the incoming beam. The sensitive detector window was protected using an additional Kapton foil (7 μm thickness). Furthermore, a protective cone ensured that particles could not contaminate the Kapton foil, and that only fluorescent light originating from the aerosol stream can reach the detector, excluding X-ray fluorescence from the stray contamination in the chamber. The SDD detector was operated using the Falcon readout electronics (manufacturer: XIA LCC, Oakland, USA) in an energy dispersive mode.

### Feasibility considerations

The spectroscopy of airborne aerosols using XAS is challenging due to the very small amount of airborne condensed matter. For example, an assumption of 3 × 10^6^ airborne particles with a radius of 0.1 μm, corresponds to an effective thickness of the solid phase of about 0.13 nm per 1 cm of pathlength. To assess the feasibility of measurements on such a thin film of an aqueous solution in transmission geometry, we first note that the X-ray absorption in water for energies below 1 keV is mostly larger than 5%, but decreases for the higher tender X-rays. Hence, the overall optical density of the aerosol is small, but could be sufficient for transmission spectroscopy at the O-K edge. However, the total absorption of a 1 M CaCl_2_ solution is about 0.4% at the Cl K edge (2822.4 eV), and about 0.2% at the Ca-K edge (4038.5 eV), with an expected XAS edge step in the order of 0.1–0.3%. Since this is too low for transmission spectroscopy and we aim for bulk spectroscopy, here we measure XAS spectra in fluorescence mode.

### XRF spectra and derivation of XAS spectra

**Fig. 3:**
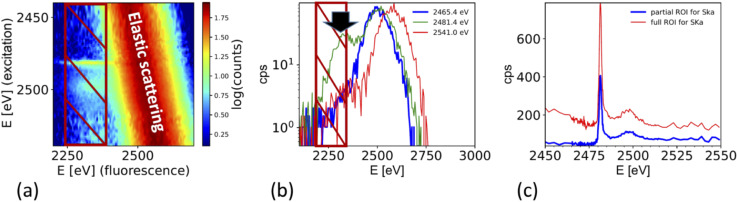
(a) X-ray fluorescence line S Kα (hatched region) and elastic scattering line for different excitation energies. (b) Cross section through (a) for different excitation energies illustrates the interference of the elastic scattering with the S Kα fluorescence. Black arrow marks the exact location of the S Kα emission line at 2308 eV. (c) Derived XAS spectrum from S Kα emission line for two cases. Case 1 ‘full ROI’ integrates over the full width of the emission line (as indicated in (a)), while the case ‘partial ROI’ integrates over the range avoiding the background by the elastic scattering as indicated in (b). The figure shows the raw data without normalization by *I*_0_. Note that (b) shows one single S XAS scan, while the data in Fig. 5 is an average of 4 single scans.

The XAS spectra shown in this paper were derived from the fluorescence spectra taken with an energy dispersive silicon drift diode. Fig. 3a shows the X-ray fluorescence spectra at different excitation energies (*y*-axis) for the case of the ammonium sulfate aerosols. Because the X-ray absorption coefficient is proportional to the intensity of the X-ray fluorescence, the intensity of the S Kα emission line (2309 eV, hatched region in Fig. 3a and b) as a function of the exciting photon energy provides a direct measure of the X-ray absorption spectrum. The most prominent feature in Fig. 3a and b is the elastic scattering peak, which has the same energy as the incoming photons. Due to the limited energy resolution of the SDD detector (∼150 eV), the elastic scattering signal partially overlaps with the sulfur emission signal and causes an unwanted background in the XANES spectra at energies below the absorption edge as shown in Fig. 3c. To minimize this effect, only the low energy part of the S Kα emission line (*i.e.* the hatched region in Fig. 3a and b) was integrated. Fig. 3b shows the S Kα emission line taken at three different excitation energies and its overlap with the elastic scattering line to illustrate the analytical challenge of the measurement. The sulfur emission line intensity is in the order of a few percent for most part of the spectrum, except where the white line peak of the XAS spectrum is (at around 2482 eV). Here, the ratio between the S Kα and the elastic scattering is only a factor of two. The elastic scattering line is also a direct measurement of the number of photons hitting the sample, and hence a good measure of the incoming photon intensity (*I*_0_) and can therefore be used to normalize the XAS spectra on *I*_0_. The effect of this procedure is illustrated in Fig. 3c. It shows the XAS spectra derived in two different ways. The XAS spectrum denoted as ‘full ROI’ (red line) is based on integrating all photon counts in the S Kα line (hatched area in Fig. 3a), while the spectrum ‘partial ROI’ uses the partial range marked as hatched area in Fig. 3b. Using the ‘partial ROI’ reduces the general noise, which is induced by the elastic scattering background. While using the ‘partial ROI’ results in a well-shaped spectrum, for the ‘full ROI’ method, the elastic scattering induces a rise in the pre-edge position towards lower energies. Such a spectrum cannot be normalized using the standard normalization procedures, and the extrapolation of the background to higher energies is not a well-known function.

### Cleaning for sample change

When changing between different samples, care was taken to thoroughly clean the system. First, the nebulizer was washed with MilliQ water, and the HPLC pump cleaned by drawing any leftover solution inside of the pump using a syringe. The aerosol system was then run with pure demineralized water to wash the pump, clean the flow system and check for a background signal. Then either the fluorescence at one energy above the edge energy, or a blank spectrum from nebulized MilliQ water was taken to assess the background contamination for the XAS measurements. Once this procedure established that the system was sufficiently clean, the MilliQ water supply was replaced by the salt solution and further XAS spectra were taken. Typically, this background is in the order of 5–10% of the true aerosol signal. The main reason for this background signal is that scattered photons excite aerosol deposits in the region of the aerosol in- and outlets.

## Results and discussion

### Stability of free-flowing aerosol stream

#### Importance of stability for XAS

**Fig. 4:**
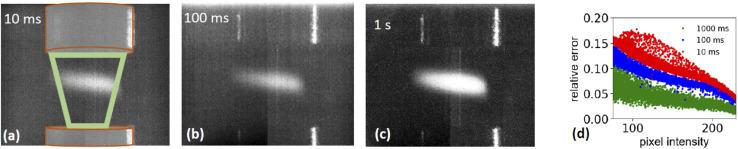
Photographs of stable aerosol flow illuminated by a laser diode and recorded with different dwell times. (a) 10, (b) 100, (c) 1000 ms. The region surrounded by the green trapezoid symbolizes the region and direction of the sample flow (d) relative error for each pixel as function of pixel intensity, calculated from repeated images during a 30 second period.

The described setup records X-ray absorption spectra (XAS) from a continuously flowing stream of aerosol particles (Fig. 4). XAS spectra are measured by scanning the photon energy of the incoming photons and recording the fluorescent light emitted by the sample. Because the fluorescence yield is proportional to both the absorption coefficient and the amount of material in the X-ray beam, meaningful spectra can only be retrieved, if the amount of sample in the beam is stable during measurement. Hence, both spatial and temporal stability of the aerosol stream is a critical boundary condition.

**Fig. 5:**
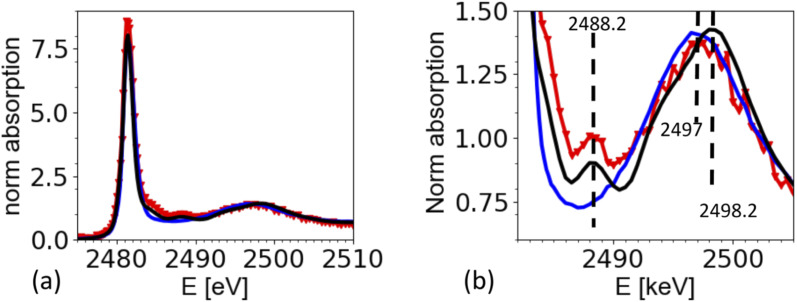
Sulfur K edge absorption spectra of ammonium sulfate aerosols (red) generated from a 0.5 M solution, compared to references of a 0.5 M ammonium sulfate solution (blue) and crystalline ammonium sulfate (black) measured in TEY. (a) Full spectrum (b) magnification in the energy range to illustrate the presence and detectability of small spectral features. For better comparison of the spectral features, the spectra were normalized such that the integrated XAS signal in the energy range 2490 to 2510 eV is equal for all spectra.

#### Stability

Fig. 4 shows the free-flowing aerosol stream illuminated by a laser diode beam of about 2 mm diameter. Only the illuminated part of the aerosol stream is visible, and the aerosol flows through the region marked by the green trapezoid. The images were taken with the endstation microscope (Leica Zoom APO 16) and a digital camera (Prosilica AV GC-D2450C). The dynamic range of the intensity was digitized in 256 steps, hereafter called ‘digitized intensity’. To assess the temporal stability of the sample in relation to different exposure times, three series of consecutive images with different exposure times, *τ*_exp_, of 10, 100, and 1000 ms, were taken. For each pixel at position (*i*, *j*) the averaged intensity *I*_*i*,*j*_ was calculated. The quantity 
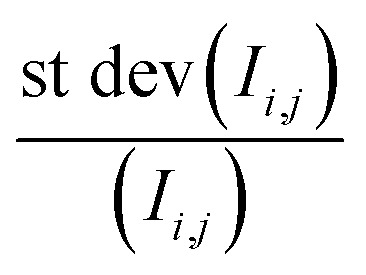
 is the relative error of the intensity in each pixel. This quantity allows to qualitatively assess the spatial sample stability of the system for a measurement which averages over timescales with exposure time *τ*_exp_. Fig. 4d shows the relative error of each pixel for three different exposure times. Here only pixel within the sample (*i.e.* with a digitized intensity < 75) and pixel below camera saturation (digitized pixel intensity < 240) were considered. Images taken at 10 ms dwell time show a typical error of more than 15%, for 100 ms it is less than 10%, and for 1 s dwell time less than 5%. These numbers indicate that integration times of at least 1 s or more are needed for sufficient statistics for each energy point when taking a XAS spectrum.

### Spectroscopy on aerosols generated from various salt solutions

To demonstrate the functionality of the method we study the drying of droplets generated from nebulized salt solutions with very different deliquescence humidity rh_del_, such as CaCl_2_ (rh_del_: 31%), (NH_4_)_2_SO_4_ (rh_del_: 81%) and KCl (rh_del_: 84%).^71,72^ The salt solutions were nebulized with dry nitrogen and sent through the dryer (residence time 1–2 s). The relative humidity at the exit of the dryer was not monitored during the experiment, but was tested beforehand and was in the order of 10–30% rh, and hence below the deliquescence humidity of all used salts.

The production and drying process occurs in 2 steps: first, the solution is nebulized with dry nitrogen. In a second step, after travelling through the tubing for about 0.4–1 second, the airborne droplets are dried. The changes in humidity between nebulization and drying steps can be estimated from the gas and liquid flow rate at the nebulizer entry. Assuming that pure water is nebulized with a liquid flow rate as low as 0.035 ml min^−1^ into a N_2_ gas flow of 1.8 l min^−1^, the relative humidity would be 100%, if all water is completely evaporated into the gas phase. In the present experiment a much higher liquid flow rate of 0.6 ml min^−1^ is used. Then only 6% of the droplet mass can evaporate, before the gas is saturated with water vapor (*i.e.* 100% humidity is reached). This implies that the salt concentration of the droplets when entering the dryer is only slightly, by less than 6%, enhanced. When travelling through the dryer the humidity is reduced to about 10–30% rh within the residence time of about 1–2 seconds in the dryer. This process is fast, because there is practically no diffusive transport limitation in the gas phase or in the droplet on a timescale of seconds. For a typical liquid phase diffusion constant of *D* = 10^−9^ m^2^ s^−1^, the characteristic diffusion length for timescales of 1 second is (*D*_*t*_)^−1/2^ = 10 μm, which is large compared to the droplet size. Therefore droplets of (NH_4_)_2_SO_4_ or KCl solutions will quickly dehydrate and most likely crystalize. For CaCl_2_ solution droplets, the situation can be different because the deliquescence humidity for CaCl_2_ of 31% rh, which is only slightly above the lowest achievable humidity in the dryer, and the nucleation of a crystalline phase may be kinetically hindered. Therefore, the system can be forced either into supersaturated droplets, crystalized CaCl_2_, or a CaCl_2_ hydrate.

### Ammonium sulfate containing aerosol

The spectrum of dissolved ammonium sulfate is very similar to the one of crystallized ammonium sulfate in Fig. 5a. There is a strong white line at 2481.3 eV, and a wide peak in the region from 2495 eV to 2502 eV. The maximum of this band is at 2497 eV for the aqueous solution, and at 2498.2 eV for the solid reference, which also shows a small peak at 2488.2 eV, which is the characteristic peak of for the crystallized ammonium sulfate crystal structure. The spectrum from the airborne particles shows the characteristic peak for crystalline ammonium sulfate at 2492.2 eV. Interestingly, the wide band in the region 2495 eV to 2502 eV, appears to be closer to the spectrum of the aqueous solution of ammonium sulfate. The complete analysis is beyond the scope of this paper, but we speculate that remaining water in the crystal may cause this shift. This would be in line with the observation by Zelenay^36^ and co-workers, who also found that O-K edge XANES spectra for drying ammonium sulfate, can be in between the ones for an aqueous solution and fully crystalized matter.

Sumarizing, the spectrum taken from the aerosols is consistent with the one from the solid references. The spectra show that the drying of the aerosol particles indeed caused the rapid crystallization of ammonium sulfate from the airborne microdroplets. We note that the strong presence of elastic scattering hampers the normalization of the spectra, which may be one reason why the intensity of the white line at 2481.3 eV seems higher for spectra taken from aerosols compared to the one taken from the references. In general, line intensities in XAS can be affected by normalization artifacts or over-absorption, in contrast to peak positions and spectral features. We have tried different normalization parameters, leading to different results for the white line intensity, but keeping all structural features the same.

### Calcium chloride containing aerosol

#### Ca K-edge spectra

**Fig. 6:**
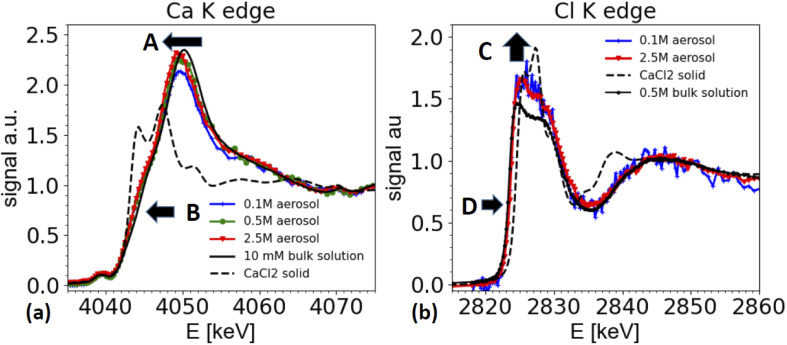
XAS spectra from nebulized solutions with different CaCl_2_ starting concentrations after passing the dryer and comparison with spectra taken from bulk solutions and solid references. (a) Ca K-edge spectra from airborne aerosols, generated from nebulized solutions of 0.1 M, 0.5 M and 2.5 M CaCl_2_ (blue, green, red lines, respectively), compared with a spectrum from a 0.5 M CaCl_2_ solution (black), and solid CaCl_2_ (dashed black line) measured in TEY mode. Arrow A marks the shift of the white line energy of aerosol samples relative to the dilute bulk solution. Arrow B marks the enhanced shoulder region for aerosol samples. (b) Cl K edge spectra from airborne aerosols generated from a CaCl_2_ solution of 0.1 M, and 2.5 M solutions (blue and red lines, respectively) compared with a XAS spectrum taken from a 0.5 M bulk solution (black line, corrected for over-absorption) and CaCl_2_ solid (dashed line). Arrow D points towards a slight shift of the edge position of the aerosol sample compared to the solution of a dilute reference. The data for the Ca K-edge spectra were smoothed using a boxcar smoothing of 2 bin width.

Fig. 6 compares XAS spectra taken from 3 different aqueous CaCl_2_ solutions (0.1 M, 0.5 M and 2.5 M), with a reference spectrum taken from solid CaCl_2_ (dashed line in Fig. 6a in TEY), and a dilute bulk solution (10 mM CaCl_2_). Clearly, the aerosol spectra resemble the solution spectrum, and not the one of a solid. The main white line position is shifted towards lower energies when compared with the spectra of a dilute solution (marked as A in Fig. 6a).

Furthermore, the edge of the aerosol spectra (see arrow marked as B in Fig. 6a) shows a shoulder, which is less marked in the spectra of a dilute solution. This observation is consistent with the work by Fulton and co-workers,^73,74^ who compared a diluted solution with Ca^2+^ ions with a 6 M solution of CaCl_2_. In this work also the white line peak shifted towards lower energies and a shoulder feature (around 4045 eV) is unfolded in the concentrated solution (2.5 M spectrum in Fig. 6a).

These spectral features contain important structural information about the coordination of the Ca^2+^ ions, and hence are relevant for the ion's chemical role. The spectra can be interpreted following Fulton and co-workers:^74^ the main peak at 4050/4049 eV is primarily due to the single scattering along the Ca–O pathway, and is characteristic for the presence of a fully hydrated Ca^2+^ ion. The 1s to 4p transition (feature B) and the slight shift of the white line is also visible in the solid CaCl_2_·6H_2_O structure (not shown, see ref. 74). Hence, the similarity of the spectra from the aerosol sample with the one of CaCl_2_·6H_2_O indicates that the aerosol samples have a fully hydrated Ca^2+^ ion, and that the Cl^−^ is present only in the second coordination shell. Therefore, the spectra from the aerosol droplets resemble the spectra of a highly concentrated CaCl_2_ solution (several M concentration). We note that the white line appears to be the highest for spectra taken from the highest starting concentrations. However, this effect could be an artifact from the elastic scattering present in the spectra, as demonstrated in Fig. 3 and in the discussion of the ammonium sulfate spectra above. This contribution is hard to quantify and increases for low concentrations and hampers the normalization procedure for XAS spectra, such that relative peak ratios can be affected. However, peak positions and shape features, such as the observed shoulder of the peaks remain unaffected.

#### Cl K-edge spectra

Fig. 6b compares the spectra from a solid CaCl_2_ reference (black dashed line), with the XAS spectra generated from nebulized dried solutions with 0.1 and 2.5 M solutions of CaCl_2_ (blue and red lines), and with a 0.5 M bulk solution of CaCl_2_ (black line). Similar to the Ca K-edge, the absorption spectra taken from aerosols are not consistent with the one of a CaCl_2_ solid reference (dashed line in Fig. 6b), but resemble the reference spectra taken from a 0.5 M CaCl_2_ solution. The XAS spectra are also very similar to the spectra of HCl solutions,^75^ where the Cl^−^/H_3_O^+^ ion pair is dominant.

Two differences can be observed in Fig. 6b. First the intensity of the ‘white line’ region line (2824–2828 eV) is slightly enhanced for the aerosol spectra compared to the one of the reference solution (marked as C in Fig. 6b). In XAS, white line intensities always need to be taken with some care, as they can be hampered by over-absorption, but also by normalization artifacts. Other than for the ammonium sulfate spectra shown in Fig. 5, here the white line intensity was insensitive to changes of normalization parameters, and hence is more reliable, but still needs to be taken with care, as it could be affected by systematic errors in the over-absorption correction for the spectrum from the 0.5 M bulk solution. Secondly, and more importantly, there is a slight shift of the edge position of the white line by about 0.3 eV towards higher energies for the spectra taken on aerosols compared to the ones of the bulk solutions (marked as D in Fig. 6b).

We note that a similar shift of the white line has also been observed by Fulton and co-worker,^75^ when comparing a 2.5 M NaCl solutions with various HCl solutions of much higher concentration. While the solutions of HCl and CaCl_2_ contain different cations, they have the same anion, namely Cl^−^. As XAS is local probe, the Cl K edge XANES spectra probe the local coordination around the Cl^−^ ion, and hence similar trends can be expected in both solutions.

Due to the water loss during dehydration of the droplets, the CaCl_2_ concentration in the droplets increases and the droplet concentration adjusts to the humidity at the exit of the dryer. In this specific experiment, we are not certain whether we really reached a relative humidity below the deliquescence humidity (32% rh) of CaCl_2_, as the humidity at the dryer's exit was not measured, and the formation of liquid water was observed in the entry region of the dryer. Hence, we cannot be certain whether the solution in the droplets is indeed supersaturated. However, the signature of the XAS spectra are clearly consistent with a very concentrated solution.

### Nebulized KCl solution and CaCl_2_/KCl mixed solution

**Fig. 7:**
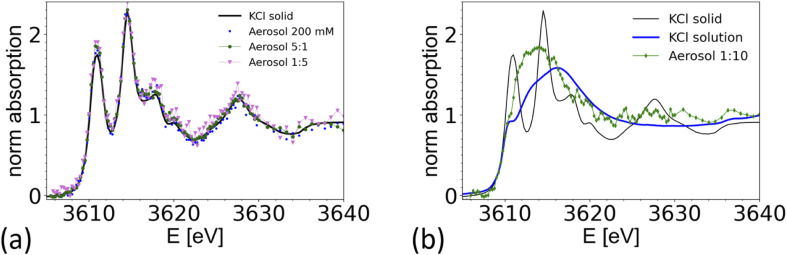
Comparison of K-K edge XAS spectra taken from aerosol generated from various KCl/CaCl_2_ aqueous solutions compared with references. (a) XAS spectra from aerosols generated from a pure 0.2 M aqueous KCl solution (blue dots) and solutions with different K/Ca ratio; green dots: K/Ca = 5 : 1, with 0.5 M KCl; magenta K/Ca = 1 : 5 KCl with 0.1 M KCl, compared with a spectrum of crystalline KCl (black line) measured in TEY mode. (b) XAS spectrum taken from a 1 : 10 K/Ca solution with 0.05 M KCl (blue), compared with a spectrum from crystalline KCl, and of a 0.5 M KCl solution (corrected for over-absorption). Chamber pressure was 600 mbar, except for the 1 : 10 K/Ca solution, where the pressure was 500 mbar. Elemental ratios refer to molar ratios.

To demonstrate the feasibility of studying mixed systems, an aerosol was generated from a pure KCl solution (0.2 M) and from mixed KCl/CaCl_2_ solutions of different K/Ca molar ratio ranging from 5 : 1 (0.5 M of KCl) to 1 : 10 (0.05 M of KCl). The results are summarized in Fig. 7 and compared with a XAS spectrum taken from crystalline KCl. For aerosols generated from a pure KCl solution and from the 5 : 1 and 1 : 5 solutions, the XAS spectrum are consistent with the one of crystalline KCl. Hence the particles quickly crystalize within 1–2 seconds after passing the dryer. The timescale is estimated from the gas flow rate, tube length and diameter. This observation is consistent with the known deliquescence humidity for KCl of 85%,^76^ which is well above the humidity at the dryer's exit.

Interestingly, the spectrum of an aerosol nebulized from a solution with a 1 : 10 KCl/CaCl_2_ ratio does not show the characteristic peaks of crystalline KCl, as illustrated in Fig. 7b. The spectrum appears featureless, as typical for the spectra of ions in amorphous or aqueous environments. However, the main peak is around 3614 eV, about 3 eV below the maximum intensity found in the reference spectra from a 0.5 M KCl solution, making the spectrum also inconsistent with the one of a 0.5 M KCl solution. A detailed analysis of this spectrum is beyond the scope of this paper. We note that Deng and coworkers,^77^ studied the formation of CaCl_2_ and KCl in concentrated mixed solution, and reported the presence of crystalline KCl for a K/Ca ratio of 0.1, but not for K/Ca of 0.07 and lower, which indicate that KCl either does not form in the presence of concentrated CaCl_2_, or that its formation is kinetically hindered. This example shows that it is possible to spectroscopically analyze the chemical structure of mixed systems in airborne particles, where the smaller component is in the order of 10% of the total material.

## Conclusion

### Proof of concept for aerosol spectroscopy

A new setup for performing X-ray absorption spectroscopy in the tender X-ray regime on a free-flowing stream of airborne aerosol particles is presented. As proof of concept, a simple system was employed involving the crystallization of inorganic salts from nebulized solution droplets. The sensitivity of the system was found to be sufficient for studying pure airborne particles. In addition, the findings show that it is feasible to measure XAS spectra from components in the aerosol particles, if they are in the order of 10% of the main aerosol content. Typical integration times for the spectra shown were in the order of 1–3 hours.

### Complementarity to aerosol lens systems

The presented approach is complementary to systems which employ aerosol lenses to inject the aerosol as a thin jet into a low-pressure chamber, which may change the thermodynamic state of the matter during its flight through the aerosol lens. For operation of an aerosol lens system, in particular when connected to XPS^51^ low pressures are needed. For XAS at tender X-rays, this boundary condition can be relaxed as the photon absorption in the gas phase is sufficiently low if helium is used. The presented experiments were performed at the same pressure in the aerosol flow system from sample preparation, during chemical processing in the flow system, all the way down to the point of spectroscopic analysis. Hence, the thermodynamic state of the aerosol particles can be controlled during the whole process from sample generation to spectroscopic analysis.

### Potential future applications in atmospheric sciences

The presented experimental approach aims to perform model laboratory experiments to decipher fundamental chemical processes in airborne aerosols. As XAS probes the local coordination of atoms in matter, the physical and chemical phase state and electronic properties, one can envision a wide range of applications in many scientific fields. For example, the technique could address important questions related to the chemistry of atmospheric aerosols, such as the study of the oxidation state of aerosol constituents in redox systems. Another important application is the *in situ* study of the phase state of non IR active inorganic matter in mixed organic aerosols. Here, *in situ* XAS experiments could directly probe the chemical state of the inorganic fraction in the aerosols. Moreover, photochemistry could be another important potential research field, where XAS could be applied to study photochemical processes in aerosols. With a residence time of individual droplet in the order of 1–2 ms in the measurement region, photochemical processes faster than 1 ms could be studied by direct illumination of the sample with light, using pump probe data acquisition schemes. For the study of slower systems, a photochemical reactor with longer residence time could be added into the flow system to process the aerosol prior to measurement.

### Nucleation studies

The described experiment opens a novel avenue to study supersaturated systems, or the nucleation of new phases. This question is obviously highly relevant to atmospheric sciences, but also to fundamental questions in synthetic chemistry. Nucleation studies can be seriously hampered by sample container interfaces, which may induce heterogeneous nucleation. Once nucleation occurs in a bulk sample, often rapid growth of the freshly nucleated new phase occurs, which makes it difficult to study the very first moments and products of the nucleation process. Such problems can be solved by using a constant stream of airborne particles or droplets, which serve as a large number of identical chemical ‘micro-reactors’. With all airborne droplets suffering equal chemical treatment, such as drying, the addition of gases which may induce crystallization, or cooling, one would generate statistically relevant averaged information about the system's behavior. As the aerosols are small there would be practically no diffusive barriers inside of the aerosol droplets, and with gas flow speeds of more than 1 m s^−1^ in tubes, and 1 m distance between the region where the crystallization is induced, typical timescales of 1 second or less can be studied, in an experiment operating with a steady state gas flow. With residence time of milliseconds for individual particles in the measurement region itself, also shorter timescales of some 10 ms can be envisioned, if the zone where nucleation is induced is brought close to the measurement zone. Currently, the experiment is operated at an ambient pressure of 500–700 mbar of helium, which would fundamentally allow to study even the oxygen K edge (543.1 eV). Hence, it would be conceivable to further develop the setup and to study the effect of impurities on ice nucleation. While atmospheric science is one of the key applications for this instrumentation, applications in other fields are conceivable, in particulate in liquid phase, nanoparticle and synthetic chemistry.

## Author contributions

TH: conceptualization, investigation, formal analysis, methodology, funding acquisition, supervision, writing-original draft, instrument development; CB: investigation, writing-review & edit, formal analysis, visualization; JMX: investigation, writing-review & edit, instrument development; MHR: investigation, writing-review & edit, formal analysis, instrument development.

## Conflicts of interest

There are no conflics of interest to declare.

## Supplementary Material
